# COVID-19 and Seasonal Adjustment

**DOI:** 10.1007/s41549-022-00071-z

**Published:** 2022-07-11

**Authors:** Barend Abeln, Jan P. A. M. Jacobs

**Affiliations:** 1 Bussum, The Netherlands; 2 CAMA and CIRANO, Faculty of Economics and Business, University of Groningen, P.O. Box 800, 9700 AV Groningen, The Netherlands

**Keywords:** COVID-19 crisis, Seasonal adjustment, Real GDP, Initial claims, Revisions, C22, E24

## Abstract

In this paper we study the impact of COVID-19 on seasonal adjustment. We focus on whether special adjustments are required to treat the COVID-19 crisis as an outlier as suggested by Eurostat in the application of three seasonal adjustment procedures: X13-ARIMA-SEATS, the industry standard, Seasonal-Trend decomposition based on Loess (STL) and a new method CAMPLET, a acronym of its tuning parameters. In addition we investigate whether revisions occur. We show results of seasonal adjustments for the quarterly series real GDP in the Netherlands, and the weekly series U.S. Initial Claims. Seasonal adjustment with X13-ARIMA-SEATS and CAMPLET requires modifications in the implementation of the standard procedure to treat the COVID-19 crisis as an outlier; STL can be applied straightforwardly. Differences in seasonally adjusted values are generally small around COVID-19. Finally, X13-ARIMA-SEATS and STL seasonal adjustments are subject to revision, which probably will lead to the COVID-19 crisis becoming less deep when new observations become available.

## Introduction

Economic time series are typically seasonally adjusted before being used in economic, econometric and policy analyses, where seasonality is defined as systematic, although not necessarily regular or unchanging, intrayear movement that is caused by climatic changes, timing of religious festivals, business practices, and expectations (Hylleberg, 1986). Seasonal adjustment consists of the estimation of the seasonal component and, when applicable, also trading day and moving holiday effects, followed by their removal from the time series. The goal is usually to produce series whose movements are easier to analyze over consecutive time intervals and to compare to the trajectories of other series in order to detect co-movements (U.S. Census Time Series and Seasonal Adjustment https://www.census.gov/topics/research/seasonal-adjustment.html; Wright, 2013). For common guidelines for seasonal adjustment within the European Statistical System, see Eurostat (2015).

Several seasonal adjustment methods have been proposed, which we broadly classify in three groups. The first group is the X11 family, i.e. methods based on moving averages like X11 (see e.g. Ladiray and Quenneville, 2001), X12-ARIMA (see the appendix of Wright, 2013) and X13-ARIMA-SEATS (for details see the X-13ARIMA-SEATS Seasonal Adjustment Program homepage at the U.S. Department of Commerce Census Bureau https://www.census.gov/srd/www/x13as/), and methods based on ARIMA models like TRAMO-SEATS (Gómez & Maravall, 1996). The methods are applicable for quarterly and monthly time series. Ladiray et al. (2018) present some ideas to adapt the X11 family to weekly and daily data. A second group of methods is based on STL (a Seasonal-Trend decomposition procedure based on Loess), a non-parametric method introduced by Cleveland et al. (1990). This group of methods is quite flexible. Cleveland and Scott (2007) and Cleveland et al. (2018) perform seasonal adjustment of weekly time series building upon the seminal contribution of Pierce et al. (1984). Ollech (2021) proposes a method for daily time series based on STL. A third class of methods employs structural time series models or unobserved components models, which are also quite flexible in dealing with time series with different frequencies although calendar effects may be less easy to handle. Examples are Harvey et al. (1997), Koopman and Ooms (2003), De Livera et al. (2011), McElroy et al. (2018) and Proietti and Tommaso (2021).^1^

Recently, Abeln et al. (2019) presented a new seasonal adjustment method CAMPLET, an acronym of its tuning parameters. The method consists of a simple adaptive procedure to extract the seasonal and the non-seasonal component from an observed time series. Once this process is carried out, there will be no need to revise these components at a later stage when new observations become available. CAMPLET can be applied to quarterly, monthly, weekly and daily data.^2^

In this paper we study the impact of COVID-19 on seasonal adjustment. We focus on whether special adjustments are required to treat the COVID-19 crisis as an outlier as recommended by Eurostat (2020) to three seasonal adjustment procedures: X13-ARIMA-SEATS, STL and CAMPLET. In addition we investigate whether revisions occur when new observations become available. We apply Census X13ARIMA-SEATS (henceforth X13), the combination of Census X12-ARIMA and TRAMO-Seats which has become the industry standard, and CAMPLET, the method we proposed recently to the quarterly series of real GDP in the Netherlands. For weekly data, Lewis et al. (2021) recommend to transform series to logs, take annual or 52 weeks differences, and manual adjustment for problem weeks (moving holidays etc.). In this paper we use STL and CAMPLET for seasonal adjustment of the weekly series U.S. initial claims.

We find that seasonal adjustment with X13 and CAMPLET requires adjustments in the implementation of the standard procedure to treat the COVID-19 crisis as an outlier; STL can be applied straightforwardly. In addition we observe that differences in seasonally adjusted values around the COVID-19 crisis are small. From the analysis of the weekly series U.S. Initial Claims we learn that STL seasonal adjustments follow observed values closely, whereas CAMPLET attributes part of the increase to a change in the seasonal pattern. Seasonal adjustments of X13 and STL are subject to revision, which probably will lead to the COVID-19 crisis becoming less deep when new observations become available.

The remainder of this paper is organised as follows. Section 2 discusses seasonal adjustment methodology and describes the seasonal adjustment methods used in this paper. Section 3 provides an illustration with a quarterly and a weekly series. Section 4 concludes.

## Seasonal Adjustment Methodology

### Seasonal Decomposition

An observed time series $y_{t}$ can be decomposed into a trend-cycle $y_{t}^{tc}$, seasonal $y_{t}^{s}$, irregular $y_{t}^{i}$ component, and deterministic effects due to the number of trading days $y_t^{td}$, and holidays $y_t^{h}$, such as Easter and Christmas (Ghysels & Osborn, 2001, Section 4.2). Assuming the additive version of the decomposition, we get

1 $$\begin{aligned} y_{t}= y_{t}^{tc} + \underbrace{y_{t}^{s}}_{\text {seasonal effects}} + \underbrace{y_t^{td} + y_t^{h}}_{\text {calendar effects}} + y_{t}^{i}, \qquad t=1,\ldots ,T. \end{aligned}$$

The multiplicative decomposition yields

2 $$\begin{aligned} y_t =&\tau _t \times c_t \times s_t \times i_t, \end{aligned}$$

where $\tau _t$ is the trend, $c_t$ is the cycle, $s_t$ is the seasonal, $i_t$ is the irregular component; calendar effects have been omitted for convenience.

### Description of Methods Used in this Paper

#### X13

X13 is based on the multiplicative decomposition (2).^3^ In a *Pre-treatment* step the series is extended forward and backwards using a regression model with ARIMA residuals (a regARIMA model). In addition outliers, and trading-day and holiday effects (calendar effects) are adjusted for. The actual seasonal adjustment consists of sequential moving average filters for the components (X-11) or ARIMA model-based adjustment (SEATS).

For details see: U.S. Department of Commerce Census Bureau http://www.census.gov/srd/www/x13as/.

#### STL

STL decomposes a time series ($y_t$) additively into a trend-cycle ($\tau _t$), a seasonal ($s_t$) and an irregular component ($i_t$). The computation of each component involves a double recursive procedure: an inner loop that is used for the decomposition and an outer loop for extreme value adjustment. The inner loop consists of trend adjustment, preliminary period-wise smoothing, smoothing the preliminary seasonal component, obtaining the seasonal component, seasonally adjusting the original time series, and obtaining the trend. After the iterations of the inner loop are completed, estimates of the trend-cycle and the seasonal components are obtained, so one can estimate the irregular component. To correct for outliers, the estimated irregular series is used to calculate robustness weights, which are inserted in the next iteration of the inner loop. Incorporating these weights makes STL robust to aberrant behaviour in the data, like the COVID-19 crisis.

In STL smoothed values are computed with a locally weighted regression smoother (Loess) and moving averages. In Loess regressions a weight is attached to each observation of the time series. This weight is negatively related to the distance (in time) between a given observation and the value that is to be smoothed. If the distance is too large, the weight is zero.

For details see Cleveland et al. (1990) or Ollech (2021).

#### CAMPLET

CAMPLET is based on an additive decomposition of an observed series ($y_t$) into a non-seasonal ($y_{t}^{ns}$) and seasonal ($y_{t}^{s}$) component

3 $$\begin{aligned} y_t = y_{t}^{ns} + y_{t}^{s}, \qquad t=1,\ldots ,T. \end{aligned}$$

Differences between average values of groups of corresponding observations of a time series of a number of full years can be decomposed into seasonal and a non-seasonal components. The non-seasonal change is assumed to be on average the same between the groups of corresponding observations. If the average change of one group differs from the others, this would be a seasonal effect. The non-seasonal components constitute a linear progression, with the average non-seasonal change equal to the difference. Average seasonal components are then the differences between group averages of observed data and non-seasonal components. Note that in every period a time series has a full set of latent seasonal components.

CAMPLET seasonally adjusts a time series on a period-by-period basis. If a new observation becomes available in period $t+1$, the seasonal factor from the previous period *t* that corresponds to this observation applies. If the new observation fits the extrapolation of the linear non-seasonal progression and this seasonal factor, then the average non-seasonal change and the corresponding seasonal factor are also valid for the new observation $t+1$. The corresponding average seasonal factor of period *t* can be applied to adjust the new observation in period $t+1$. This adjustment is final: future events cannot have an impact on the decomposition of past observations. Every observation is seasonally adjusted as if it is the final observation of a time series. The seasonal and non-seasonal components are determined on the basis of the new observation and on what preceded.

A new observation does not need to fit the extrapolation of the linear non-seasonal progression and the corresponding seasonal factor of the previous observation. To adjust the new observation the change of the non-seasonal progression is updated. The difference between the observed value $y_{t+1}$ and the extrapolated value for $t+1$ based on information available in *t* is denoted by the *extrapolation error* $\hat{e}_{t+1}$. The extrapolation error in period $t+1$ is divided over changes in the seasonal and the non-seasonal in period $t+1$. The non-seasonal change $g_t$ is assumed to rotate according to $g_{t+1}=g_{t}+\hat{e}_{t+1}/\ell _{t+1}$, where $\ell _{t+1}$ is the *common adjustment length*, a parameter in CAMPLET which is assumed to be equal to $1.5 \times$ the length of the seasonal cycle. The change of the non-seasonal progression also affects the seasonal components. Once the value of the new seasonal component in period $t+1, y_{t+1}^{s}$, is known, the seasonally adjusted value $y_{t+1}^{sa}$ can be calculated from decomposition (3) and the latent seasonal components are updated.

CAMPLET needs starting values for the the seasonally adjusted value $y_{0}^{sa}$, the non-seasonal change $g_0$, and the latent seasonal components in the starting period. These can be obtained from one seasonal cycle, if there are no outliers. An outlier in the first seasonal cycle also occurs in the initial seasonal pattern. To avoid this CAMPLET is run for the first three seasonal cycles, the non-seasonal change is extrapolated backwards to the first observation, and the full series is adjusted.

The period-by-period seasonal adjustment in CAMPLET allows dealing with calendar effects, including outliers. To mitigate the effects of an outlier on the seasonal components, one can increase the adjustment length for that period, but reset it to common adjustment length of one-and-a-half cycle for the next observation. If the outlier also occurs one seasonal cycle later, the seasonal pattern is assumed to have been changed. The second time an outlier is detected, the adjustment length is shortened to one cycle to adopt the new seasonal pattern. This property of CAMPLET makes it well suited to capture breaks in seasonal patterns.

For details see Abeln et al. (2019). The package can be downloaded at http://www.camplet.net.

### Adjustments Because of the COVID-19 Crisis

In a methodological note to provide guidance on the treatment of COVID-19 crisis effects on data, Eurostat (2020) wrote:

In the context of seasonal adjustment, a calendar adjustment corresponds to a predictable and recurrent phenomenon linked to the calendar. In contrast, the COVID-19 crisis is completely different and must be handled by means of outliers. At this stage, the data point in question *shall not be treated as a seasonal outlier*, since it would imply that the current COVID-19 outbreak occurs each year in the same period with similar magnitude. For each following observation, a change may occur in the seasonal pattern and/or a discontinuity in seasonality.

As will be illustrated in the next section, X13 and CAMPLET require adjustments in the implementation of their standard procedures to treat the COVID-19 crisis as an outlier. STL, however, does not need modifications!

## Illustrations

### Data and Settings of Seasonal Adjustment Methods

Data on real GDP in the Netherlands used in this paper are from Statistics Netherlands (CBS) Statline; the U.S. Initial Claims series comes from FRED, St Louis FED. Computations are done in February 2021, with the latest vintages of data available at that date.

Seasonal adjustments are computed for the whole period the series are available unless otherwise stated. All X13 and STL seasonal adjustments are done in Eviews 11, with default settings of the procedures. CAMPLET computations are done with CampletExcel-v5s4.xlsm, also with default parameters settings.

### Results

#### Real GDP in the Netherlands

**Fig. 1:**
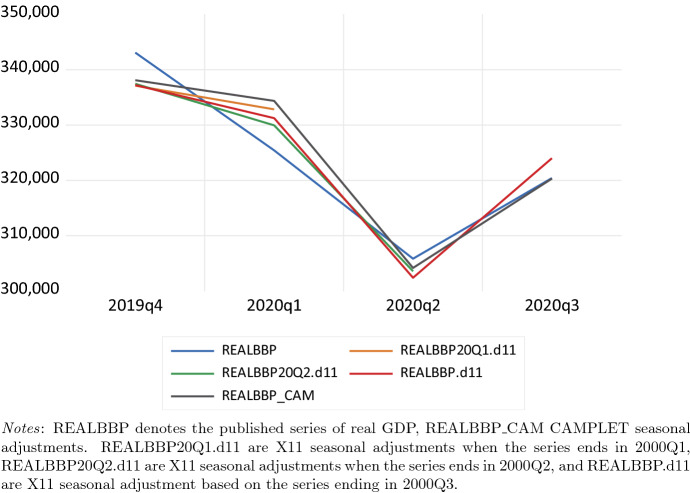
Real GDP in the Netherlands, 2019Q4–2020Q3. REALBBP denotes the published series of real GDP, REALBBP_CAM CAMPLET seasonal adjustments. REALBBP20Q1.d11 are X11 seasonal adjustments when the series ends in 2000Q1, REALBBP20Q2.d11 are X11 seasonal adjustments when the series ends in 2000Q2, and REALBBP.d11 are X11 seasonal adjustment based on the series ending in 2000Q3

Figure 1 shows the the last two years of real GDP in the Netherlands, CAMPLET seasonal adjustments, and three series of X11 seasonal adjustments, one ending in 2020Q1, one in 2020Q2 and one in 2020Q3, i.e. the quarter before the COVID-19 crisis, the COVID-19 crisis quarter, and the quarter after the crisis.

We observe that seasonally adjusted values produced by X13 and CAMPLET are close over the last year, an observation that holds for the whole series of seasonally adjusted values but is not shown here. Seasonal adjustments calculated with standard X13 and CAMPLET setting are above the observed non-seasonally adjusted value in 200Q2, the COVID-19 crisis quarter. We conclude that modifications are required to treat the COVID-19 crisis as an outlier. CAMPLET seasonal adjustments are not revised when new observations become available, whereas X13 seasonal adjustments show revisions.

#### U.S. Initial Claims

**Fig. 2:**
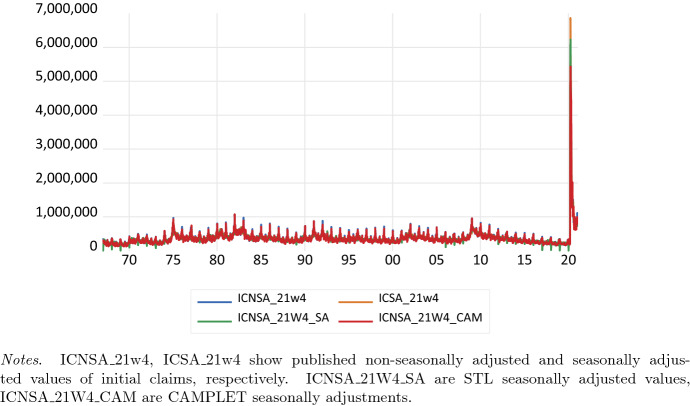
U.S. Initial claims, weekly, ending Saturday, and STL and CAMPLET seasonal adjustments, 1967W1–2021W4. ICNSA_21w4, ICSA_21w4 show published non-seasonally adjusted and seasonally adjusted values of initial claims, respectively. ICNSA_21W4_SA are STL seasonally adjusted values, ICNSA_21W4_CAM are CAMPLET seasonally adjustments

An initial claim is a claim filed by an unemployed individual after a separation from an employer. The claim requests a determination of basic eligibility for the Unemployment Insurance program. Figure 2 shows the weekly series of US initial series, based on weeks ending Saturday, and official seasonally adjusted values as available at FRED, and STL and CAMPLET seasonal adjustments for the period 1967w1–2021w4. The initial claims shows a large spike in the COVID-crisis period between March and May 2020, but STL seasonally adjusted values are close to the published non-seasonally adjusted series.

**Fig. 3:**
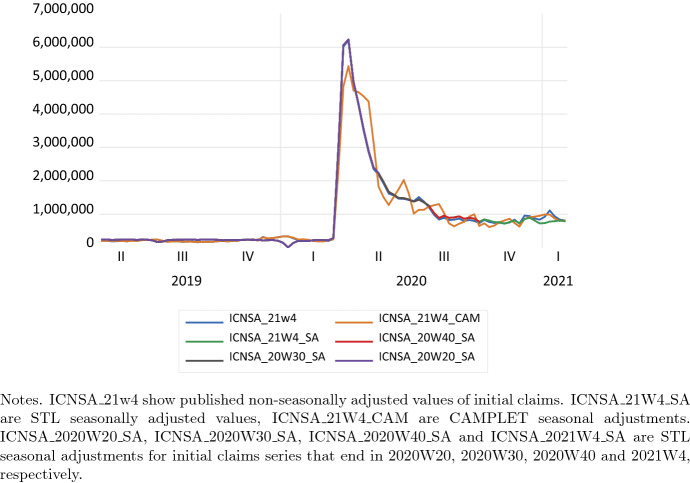
US Initial claims, STL and CAMPLET seasonal adjustments, 2019w1–2021w4. ICNSA_21w4 show published non-seasonally adjusted values of initial claims. ICNSA_21W4_SA are STL seasonally adjusted values, ICNSA_21W4_CAM are CAMPLET seasonal adjustments. ICNSA_2020W20_SA, ICNSA_2020W30_SA, ICNSA_2020W40_SA and ICNSA_2021W4_SA are STL seasonal adjustments for initial claims series that end in 2020W20, 2020W30, 2020W40 and 2021W4, respectively

In Fig. 3 we show the published non-seasonally adjusted series of initial claims, CAMPLET seasonal adjustments, and STL seasonally adjusted values for series that end in 2020W20, 2020W30, 2020W40 and 2021W4, and zoom in on the two most recent years. CAMPLET seasonal adjustments are less smooth than STL seasonal adjustment after the COVID-19 crisis.^4^ Since CAMPLET seasonal adjustments are not revised, we do not have to compute seasonal adjustments of CAMPLET for the subperiods. We observe that STL seasonal adjustments are subject to revision.

STL seasonal adjustments match non-seasonally adjusted values in the COVID-19 crisis period completely, an outcome in line with the recommendation of Eurostat (2020) discussed in Sect. 2.3 above. According to STL seasonal adjustments, there are no seasonal effects during the crisis. In contrast CAMPLET seasonal adjustments underestimate published non-seasonally adjusted initial claims in the COVID-19 crisis, suggesting a role for a seasonal effect during in the COVID-19 crisis. Hence CAMPLET has to be modified to meet the Eurostat (2000) guideline to treat the COVID-19 crisis as an outlier for this weekly series either.

### Discussion

The examples for the quarterly and weekly series illustrate that X13 and STL seasonal adjustments are subject to revisions. Revisions occur because of the pretreatment step and the seasonal decomposition in X13 and the seasonal decomposition in STL. Pretreatment, i.e. forecasting and backcasting, leads to large deviations between forecasts and realizations when a crisis like COVID-19 occurs, which necessitates treating the crisis as an outlier in the seasonal decomposition as proposed by Eurostat (2020). The use of moving averages and ARIMA models also results in revisions when new observations become available.

Realizing that after the crisis the trend/cycle and the seasonal components will be revised again, part of the impact of the crisis will no longer be attributed to the crisis dummy but to the trend/cycle or the seasonal component. Consequently the COVID-19 crisis will become less deep in due time (Abeln & Jacobs, 2021). Seasonally adjusted real GDP will be revised in upward direction and seasonally adjusted values of U.S. initial claims will be revised downwards.

## Conclusion

In this paper we study the impact of COVID-19 on seasonal adjustment. We focus on whether special adjustments are required to treat the COVID-19 crisis as an outlier in the application of three seasonal adjustment procedures: X13, the industry standard, STL and a new method CAMPLET. In addition we investigate whether revisions occur. We show results of seasonal adjustments produced with X13 and CAMPLET for the quarterly series real GDP in the Netherlands, and STL and CAMPLET seasonal adjustments for the weekly series U.S. Initial Claims.

Seasonal adjustment with X13-ARIMA-SEATS and CAMPLET requires adjustments in the implementation of the standard procedure to treat the COVID-19 crisis as an outlier as recommended by Eurostat (2000); STL can be applied straightforwardly. Differences in seasonally adjusted values are generally small around the COVID-19 crisis. Finally, X13 and STL seasonal adjustments are subject to revisions. Future revisions will probably make the COVID-19 crisis less deep.

Further research is needed to validate these findings for other series, countries and seasonal adjustment methods. To fully appreciate the recommendation of Eurostat (2020) and its consequences, simulation studies are required in which we know the processes of the components of the variables. Seasonal adjustment produces latent variables, so it makes sense to design experiments in which the processes of the non-seasonal and seasonal components are known, and possibly correlated (Hindrayanto et al., 2019).

## Footnotes

1 For other methods see references in Ladiray et al. (2018), Ollech (2021) and Proietti and Tommaso (2021).

2 Abeln et al. (2022) show how seasonal cycles with time-varying lengths can be dealt with in CAMPLET.

3 However, to deal with COVID-19 the additive decomposition is required. In September 2020 the US Department of Labor (DOL) switched from the multiplicative to the additive decomposition (Stock et al., 2021).

4 This can be easily fixed however by setting one of CAMPLET’s parameters, the multiplier *M*, which increments the adjustment length, to 100 instead of its standard value of 50.
